# Unraveling the diversity dynamics and network stability of alkaline phosphomonoesterase‐producing bacteria in modulating maize yield

**DOI:** 10.1002/imt2.260

**Published:** 2024-12-20

**Authors:** Lijun Chen, Guofan Zhu, Alberto Pascual‐Garcia, Francisco Dini‐Andreote, Jie zheng, Xiaoyue Wang, Shungui Zhou, Yuji Jiang

**Affiliations:** ^1^ Institute of Soil Science, Chinese Academy of Sciences Nanjing China; ^2^ College of Forestry Central South University of Forestry and Technology Changsha China; ^3^ College of Resources and Environment Fujian Agriculture and Forestry University Fuzhou China; ^4^ Department of Systems Biology Spanish National Centre for Biotechnology (CSIC) C/Darwin 3 Madrid Spain; ^5^ Department of Plant Science & Huck Institutes of the Life Sciences The Pennsylvania State University University Park Pennsylvania USA

## Abstract

Phosphorus, as a nonrenewable resource, plays a crucial role in crop development and productivity. However, the extent to which straw amendments contribute to the dynamics of soil alkaline phosphomonoesterase (ALP)‐producing bacterial community and functionality over an extended period remains elusive. Here, we conducted a 7‐year long‐term field experiment consisting of a no‐fertilizer control, a chemical fertilizer treatment, and three straw (straw, straw combined with manure, and straw biochar) treatments. Our results indicated that straw amendments significantly improved the succession patterns of the ALP‐producing bacterial diversity. Simultaneously, straw amendments significantly increased the network stability of the ALP‐producing bacteria over time, as evidenced by higher network robustness, a higher ratio of negative to positive cohesion, and lower network vulnerability. High dynamic and stability of ALP‐producing bacterial community generated high ALP activity which further increased soil Phosphorus (P) availability as well as maize productivity.

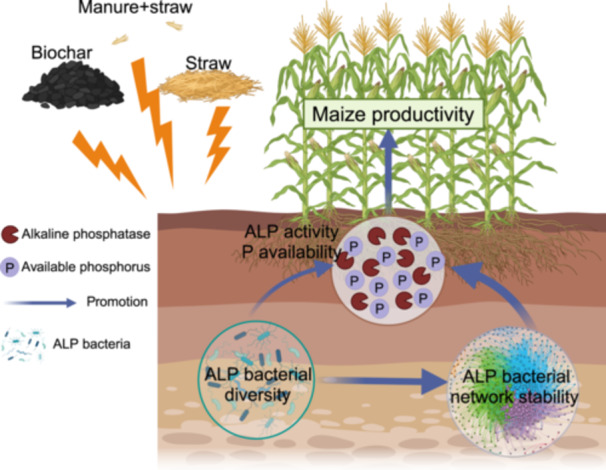

Soil bacterial community dynamically influences the soil‐plant system and regulates nutrient efficiency and plant productivity [1]. Phosphorus (P) availability in soil represents a major challenge to crop productivity, as it limits the capacity of agricultural systems to feed the world's growing population. Soil phosphorus availability can determine the growth rate and turnover of bacterial taxa, thereby influencing the temporal dynamics and structure of the bacterial community. In agroecosystems, straw amendments can boost P supply and availability (via mineralization) by regulating alkaline phosphomonoesterase (ALP)‐producing bacteria [2]. In contrast, several studies indicate that straw amendments have a neutral or even negative effect on the community diversity and structure of ALP‐producing bacteria [3, 4]. However, there have been seldom studies that have examined the dynamics of soil ALP‐producing bacterial communities over time following long‐term straw amendments.

Bacterial coexistence in complex communities results from inter‐specific interactions and the interplay of stabilizing and balancing mechanisms. Understanding the ecological mechanisms underlying spatio‐temporal turnover in bacterial communities is essential to identify the patterns of potential species interactions [5]. Principally, the stability of bacterial communities is attributed to species diversity, as there is a consensus that biodiversity has positive effects on microbial stability [6]. Previous studies have focused on understanding the intrinsic relationships between microbial diversity and stability through the development of ecological models or the resolution of simple communities [6, 7]. To date, how changes in the ALP‐producing bacterial diversity alter community stability under straw amendments has not been completely evaluated.

Soil P cycling is closely related to alterations in the diversity and network stability of the ALP‐producing bacterial community [8]. An investigation examining the temporal succession of bacterial diversity and network stability offers a theoretical foundation for understanding the mechanisms behind functional activity [9]. Different types of straw amendments (e.g., straw and straw biochar) have been used to improve P cycling dynamics by regulating the ALP‐producing bacterial community and increasing ALP activity [2]. As such, research on how the succession of specific ALP‐producing bacterial populations varies in response to different straw amendments can provide essential insights into microbial P metabolic activity [10]. Nevertheless, it remains unclear how the temporal dynamics of bacterial diversity and network stability regulate soil nutrient balances.

Here, we evaluated the temporal dynamics of ALP‐producing bacterial (*phoD* gene) diversity and network stability on an annual time scale and linked the observed patterns to their influences on soil P dynamics under diverse straw amendments. Specifically, we observed a field experiment continuously for 7 years to examine the annual succession of the ALP‐producing bacterial communities with or without straw amendments. Particularly, this study aimed to investigate (i) how and to what extent different straw amendments affect the temporal turnover of ALP‐producing bacterial community; (ii) how the observed patterns in the diversity of ALP‐producing bacterial community connect with ecological succession and network stability; and (iii) how the diversity and stability of ALP‐producing bacterial community contribute to soil P cycling dynamics and maize yield.

## RESULTS AND DISCUSSION

### Temporal dynamics of soil properties and ALP‐producing bacterial diversity

Overall, the average values of soil organic carbon (SOC), total nitrogen (TN), total phosphorus (TP), total potassium (TK), available phosphorus (AP), and available potassium (AK) significantly (*p* < 0.05) increased over time under fertilization treatments (chemical fertilizer (N), chemical fertilizer application with straw (NS), chemical fertilizer application with straw and pig manure (NSM), and chemical fertilizer application with straw biochar (NB)). compared to no fertilizer treatment (CK) (Figure S1). The ALP‐producing bacterial diversity, was calculated by Shannon index (*r* ^2^ = 0.10–0.93) and Chao1 richness (*r* ^*2*^ = 0.17–0.91), significantly (*p* < 0.001) increased over time with three straw amendments (NS, NSM, and NB) compared with CK and N treatments (Figure S2). The slopes of Shannon index (slope = 0.29–0.37) and Chao1 richness (slope = 47.94–78.67) concerning the ALP‐producing bacterial community under NS, NSM, and NB treatments were significantly (*p* < 0.05) higher compared to those in N treatment (slope = 0.14 and 22.87, respectively), primarily after the third year (Figure S2, Table S1). The importance of soil properties and bacterial niche for the ALP‐producing bacterial diversity was examined using Spearman correlation and forward stepwise regression (Figure S2). Soil fertility has been reported to promote greater microbial coexistence by enhancing niche structure and environmental heterogeneity. Straw amendments increased the heterogeneity of the soil environment, ultimately leading to higher species diversity over time [11]. These were further confirmed by the niche breadth index (*B*_*n*_) and niche overlap index (*O*_*n*_) analysis, straw amendments displaying steeper slopes of *B*_*n*_ (*r* ^*2*^ = 0.61–0.86) and *O*_*n*_ (*r* ^2^ = 0.47–0.72) compared with CK and N (Figure S3).

### Temporal dynamics of ALP‐producing bacterial community composition

The ALP‐producing bacterial operational taxonomic units (OTUs) were largely associated with Alphaproteobacteria (34.5%), Betaproteobacteria (31.0%), and Actinobacteria (25.2%) (Figure S4). The high relative abundance in these taxa was most likely due to the dynamic processing and stabilization of organic matter under straw amendments. For example, previous studies have shown alkyl carbons, aromatic C, and aromatic C‐O are released when the maize straw decomposes [12], and these molecules were effectively processed by taxa within Alphaproteobacteria. The genus of ALP‐producing bacterial community was dominated by *Bradyrhizobium* (27.7%), *Collimonas* (23.3%), and *Amycolatopsis* (10.3%) (Figure S4).

Principal coordinate analysis indicated significant (*p* < 0.01) differences in the structure of the ALP‐producing bacterial community, across fertilization treatments and time points (Figure S5). Straw decomposition may promote certain oligotrophic taxa (e.g., *Agromonas oligotrophica*) due to the release of refractory organic acid compounds such as ferulic acid, coumaric acid, and anisic acid [13, 14]. The fuzzy c‐means clustering algorithm showed significantly (*p* < 0.05) higher number of OTUs changes in NS (678), NSM (711), and NB (707) treatments compared with CK (475) and N (204) (Figure S5). Manure, straw, and straw biochar differ in the quality of organic carbon and are expected to have different impacts on soil microbial activities [15], therefore the response of the ALP‐producing bacterial community shows inconsistent tendencies under NS, NSM, and NB treatments.

### Temporal dynamics of structure and stability of ALP‐producing bacterial network

**FIGURE 1:**
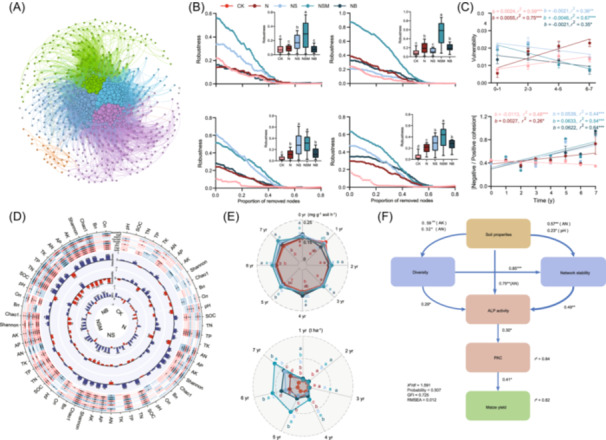
Temporal dynamics of the soil alkaline phosphomonoesterase (ALP) producing bacterial community, and its relation to ALP activity and maize yield. (A) Soil ALP‐producing bacterial networks. A connection stands for a strong (Spearman's *r* > 0.8 or <−0.8) and significant (*p*‐value < 0.01) correlation for field experiments samples. The size of each node is proportional to the number of connections. The nodes and edges were colored by module clusters. (B) Robustness is measured as the taxa are randomly removed from each network. Bars with different lowercase letters are significantly (*p* < 0.05) different by Tukey's post hoc tests. (C) Temporal dynamics of network vulnerability and values of |Negative cohesion:Positive cohesion| under different treatments. Network vulnerability is measured by the maximum node vulnerability in each network. (D) Pearson correlations between network topological properties and soil properties. (E) Temporal dynamics of soil ALP activity and maize yield. (F) Soil properties include pH, SOC, AN, and AK, while the ALP‐producing bacterial community includes diversity (Shannon index and Chao1 richness) and network stability (robustness, vulnerability, and NPC ratio). Blue and red lines indicate positive and negative effects, respectively. The width of the arrows indicates the strength of significant standardized path coefficients. Paths with nonsignificant coefficients are presented as gray lines. AK, soil available potassium; AN, soil available nitrogen; AP, soil available phosphorus; *avgK*, average degree; *avgCC*, average cluster coefficient; *B_n_*, niche breadth index; NPE, the ratio of negative to positive edges; NPC, the ratio of negative to positive cohesion; *O_n_*, niche overlap index; SOC, soil organic carbon; TP, soil total phosphorus; TN, soil total nitrogen; TK, soil total potassium. CK, no fertilizer; N, NPK fertilizer; NS, NPK fertilizer application with straw; NSM, NPK fertilizer application with straw and pig manure; NB, NPK fertilizer application with straw biochar. *p < 0.05, **p < 0.01, ***p < 0.001.

We observed that the co‐occurrence networks of ALP‐producing bacteria were modulated by straw amendments, as demonstrated by various topological parameters (Figures 1A, S6 and Table S2). The numbers of total nodes, positive and negative edges were higher in NS, NSM, and NB treatments than CK and N over time (Table S2). Furthermore, we estimated the network stability of the ALP‐producing bacterial community by analyzing network robustness (natural connectivity), vulnerability, and NPC ratio. Specifically, the 4−5 year and 6−7 year networks showed significant (*p* < 0.05) increase in natural connectivity under NS and NSM treatments compared to CK and N treatments, while vulnerability followed the opposite trend (Figures 1A−C, S6). Microbial communities with high species may exhibit greater functional redundancy, ultimately increasing the overall stability of the soil ecosystem in the face of environmental perturbations [16].

The NPC ratios were significantly (*p* < 0.05) increased under NS, NSM, and NB treatments compared with N treatment over time (Figure 1C), indicating that negative cohesions (rather than positive cohesions) dominated the network stability under straw amendments. ^18^O‐H_2_O DNA stable‐isotope probing (DNA‐SIP) microcosm experiments determined that 60.8%–69.3% and 58.7%–73.1% of the nodes in the 0–1 year and 6–7 year networks were detected, and confirmed the increase in diversity, NPC ratio, and natural connectivity under straw amendments (Figure S7). Furthermore, high proportions of negative cohesion under straw amendments suggested the potential competition of bacteria for resources or niche differentiation. Negative associations, indicated by the NPC ratio, have the potential to stabilize communities by balancing the fitness and abundance of associated individuals, thereby eliminating their dependence on interaction partners in positive feedback loops [17]. We revealed that SOC, TP, AP, AK, the Shannon index and Chao1 richness of ALP‐producing bacterial diversity, and *O*_*n*_ were significantly (*p* < 0.05) associated with network topological parameters, including total edges (*r* = 0.388–0.933), average path length (*r* = −0.889–0.671), density (*r* = −0.456–0.873), *avgK* (*r* = −0.693–0.896), and modularity (*r* = −0.718–0.853) under NS, NSM, and NB treatments (Figure 1D). Since greater niche overlap is predicted to intensify the strength of interspecies interactions [18], we suggested that straw amendments enhanced the diversity of the ALP‐producing bacterial community by boosting potential competition and niche overlap among taxa. The diversity of ALP‐producing bacteria was found to be positively (*p* < 0.05) correlated with network robustness and NPC ratio, but negatively (*p* < 0.05) correlated with vulnerability under NS and NB treatments (Figure 1D).

### Relationships of ALP‐producing bacterial diversity and network stability with soil P dynamics and maize yield

Soil acid phosphomonoesterase and ALP activities were significantly (*p* < 0.05) increased under NS and NSM treatments compared to CK after the fourth year, and the same as phosphorus activation coefficient (PAC) (Figures 1E, S8). While maize yields were significantly (*p* < 0.05) higher under NSM, NB, and NS than CK and N treatments after the fourth year (Figure S8). Random forest modeling determined that the diversity (Shannon index and Chao1 richness) and network stability (robustness, vulnerability, and NPC ratio) of the ALP‐producing bacterial community, and *O*_*n*_ contributed significantly (*p* < 0.05) to PAC (5.8%–14.0%) and maize yield (8.5%–14.5%) (Figure S9). High community diversity often coincides with greater efficacy in nutrient cycling and plant productivity due to their metabolic versatility and redundancy [19].

Structural equation modeling indicated that soil properties displayed significantly (*p* < 0.05) negative correlations with the diversity of ALP‐producing bacteria under CK and N treatments (Figures 1F, S10). There was no significant association between the diversity and network stability of ALP‐producing bacteria and maize yield through ALP activity and PAC under N treatment. In contrast, soil properties exhibited significantly (*p* < 0.05) positively correlated with the diversity and network stability of ALP‐producing bacteria under NS and NSM treatments. Notably, the diversity of ALP‐producing bacteria was positively correlated with network stability (*r* = 0.296–0.850, *p* < 0.05) under NS, NSM, and NB treatments (Figures 1F, S10). Community stability has been determined to govern the direction and intensity of relationships between biodiversity and ecosystem functioning [20]. A stable community experiences less variation in composition or function during perturbations, enabling efficient resource exchange and functional complementation in a fluctuating environment [16]. These results were consistently demonstrated further by the DNA‐SIP microcosm experiments (Figure S11). Furthermore, the ALP‐producing bacterial diversity and network stability showed positive correlations with maize yield by influencing ALP activity and PAC under three straw amendments. Although there were numeric researches focus on straw amendments impacts on soil nutrients cycling, our study estimated the potentials of network stability in regulating the connections between the bacterial diversity and ecosystem functioning using a long‐term experiment combined with ^18^O‐DNA‐SIP incubation.

## CONCLUSION

We found that management practices should prioritize the value of soil biodiversity, particularly soil microbial network stability, for promoting and maintaining nutrient dynamics and maize yield in highly disturbed agriculture ecosystems. Straw amendments led to higher rates in dynamic patterns of diversity and network stability in ALP‐producing bacteria. The combination of bacterial diversity and network stability contributed to a metabolic elevation in ALP activity that underlies microbial community interaction patterns, thereby improving maize yield. Taken together, our results offer novel insights into how the interannual succession of network stability mediates bacterial diversity and P dynamics associated with maize yield under straw amendments. Further studies should focus more on the impacts of microbial community diversity and stability on soil health and crop productivity at large spatio‐temporal scales and across different soil types.

## METHODS

Detailed procedures for sample collection, sequencing protocol, data processing techniques for sequencing data, bioinformatic and statistical analysis approaches are available in the Supplementary Information.

## AUTHOR CONTRIBUTIONS

**Lijun Chen**: Writing—review and editing; writing—original draft; visualization; formal analysis; data curation; methodology; funding acquisition; conceptualization. **Guofan Zhu**: Writing—original draft; formal analysis; resources. **Alberto Pascual‐Garcia**: Writing—review and editing; supervision. **Francisco Dini‐Andreote**: Writing—review and editing; supervision. **Jie Zheng**: Writing—original draft; formal analysis; resources. **Xiaoyue Wang**: Writing—review and editing; methodology; investigation; supervision. **Shungui Zhou**: Writing—review and editing; supervision; methodology. **Yuji Jiang**: Writing—review and editing; supervision; resources; project administration; methodology; investigation; funding acquisition; data curation; conceptualization.

## CONFLICT OF INTEREST STATEMENT

The authors declare no conflicts of interest.

## ETHICS STATEMENT

No animals or humans were involved in this study.

## Supporting information


**Figure S1.** Temporal dynamics of soil chemical properties over time under different fertilization treatments.
**Figure S2.** Temporal dynamics of ALP‐producing bacterial diversity over time under different treatments.
**Figure S3.** Temporal dynamics of niche breadth index and niche overlap index over time under fertilization different treatments.
**Figure S4.** Relative abundance of alkaline phosphomonoesterase (ALP) producing bacterial community over time under different treatments.
**Figure S5.** The structure of alkaline phosphomonoesterase (ALP) producing bacterial community.
**Figure S6.** Temporal dynamics of alkaline phosphomonoesterase (ALP) producing bacterial networks.
**Figure S7.** The co‐occurrence patterns and network stability of soil alkaline phosphomonoesterase (ALP) producing bacteria under fertilization treatments at 0‐1 and 6‐7 years in DNA‐SIP microcosm experiments.
**Figure S8.** Temporal dynamics of soil acid phosphomonoesterase (ACP) and phosphorus activation coefficient (PAC).
**Figure S9.** Random forest model on soil phosphorus activation coefficient and maize yields.
**Figure S10.** The structural equation modeling under different treatment.
**Figure S11.** The diversity of soil alkaline phosphomonoesterase (ALP) producing bacteria and its correlations with network stability in DNA‐SIP microcosm experiments.

**Table S1.** Significance comparisons of the slopes of alkaline phosphomonoesterase (ALP) producing bacterial diversity under different treatments based on a pairwise *t*‐test.
**Table S2.** The topological properties of alkaline phosphomonoesterase producing bacterial networks over time under different treatments.

## Data Availability

The data that support the findings of this study are available on request from the corresponding author. The data are not publicly available due to privacy or ethical restrictions. All sequencing reads of the ALP‐producing bacterial communities including the field and microcosm experiments have been deposited in the NCBI Sequence Read Archive under accession numbers SAMN38476769‐SAMN38476948 (Bioproject PRJNA1046151, https://www.ncbi.nlm.nih.gov/bioproject/PRJNA1046151/). The data and scripts used are saved in GitHub (https://github.com/LijunChen-88/Network-stability). Supplementary Materials (methods, figures, tables, graphical abstract, slides, videos, Chinese translated version and update materials) may be found in the online DOI or iMeta Science http://www.imeta.science/.
