# Supplementary material for: Unraveling the diversity dynamics and network stability of alkaline phosphomonoesterase‐producing bacteria in modulating maize yield
Source: Imeta. 2024 Dec 20;3(6):e260. doi: 10.1002/imt2.260 (PMC11683463; doi:10.1002/imt2.260)
Supplement: Supplementary file 1 — Figure S1. Temporal dynamics of soil chemical properties over time under different fertilization treatments. Figure S2. Temporal dynamics of ALP‐producing bacterial diversity over time under different treatments. Figure S3. Temporal dynamics of niche breadth index and niche overlap index over time under fertilization different treatments. Figure S4. Relative abundance of alkaline phosphomonoesterase (ALP) producing bacterial community over time under different treatments. Figure S5. The structure of alkaline phosphomonoesterase (ALP) producing bacterial community. Figure S6. Temporal dynamics of alkaline phosphomonoesterase (ALP) producing bacterial networks. Figure S7. The co‐occurrence patterns and network stability of soil alkaline phosphomonoesterase (ALP) producing bacteria under fertilization treatments at 0‐1 and 6‐7 years in DNA‐SIP microcosm experiments. Figure S8. Temporal dynamics of soil acid phosphomonoesterase (ACP) and phosphorus activation coefficient (PAC). Figure S9. Random forest model on soil phosphorus activation coefficient and maize yields. Figure S10. The structural equation modeling under different treatment. Figure S11. The diversity of soil alkaline phosphomonoesterase (ALP) producing bacteria and its correlations with network stability in DNA‐SIP microcosm experiments. [file IMT2-3-e260-s002.docx]

**Supplementary materials to**

**Unraveling the diversity dynamics and network stability of alkaline phosphomonoesterase producing bacteria in modulating maize yield**

**Running Title:** An insight view of bacterial network stability on maize yield

Lijun Chen^1,2#^, Guofan Zhu^1,3#^, Alberto Pascual-Garcia^4^, Francisco Dini-Andreote^5^, Jie zheng^1^, Xiaoyue Wang^1^, Shungui Zhou^3^, Yuji Jiang^1, 3^*

^1^ Institute of Soil Science, Chinese Academy of Sciences, Nanjing 210008, China

^2^ College of Forestry, Central South University of Forestry and Technology, Changsha 410004, China;

^3^Fujian Provincial Key Laboratory of Soil Environmental Health and Regulation, College of Resources and Environment, Fujian Agriculture and Forestry University, Fuzhou 350002, China

^4^Department of Systems Biology, Spanish National Centre for Biotechnology (CSIC) C/ Darwin 3, Madrid 28049, Spain

^5^Department of Plant Science & Huck Institutes of the Life Sciences, The Pennsylvania State University, University Park, Pennsylvania 16802, USA

# These authors contributed equally: Lijun Chen, Guofan Zhu.

*Correspondence: yjjiang@fafu.edu.cn (Yuji Jiang).

**METHODS**

**Field experiment**

The long-term field experiment was conducted at Yingtan Agricultural Ecosystem Experimental Station of the Chinese Academy of Sciences (116°55’E and 28°15’N) in Jiangxi Province, China. The study region is characterized by a subtropical monsoon climate, boasting an annual temperature of 17.8°C and precipitation of 1795 mm. The experimental system, established in 2010, featured plots of 20 m length × 5 m width. The soil is classified as a typical Paleudults (Ultisol, quaternary red clay as its parent material) according to the classification system of the United States Department of Agriculture (USDA). The field experiment was conducted based on a completely randomized design including five treatments with triplicates: no fertilizer control (CK), chemical fertilizer (N), chemical fertilizer application with straw (NS), chemical fertilizer application with straw and pig manure (NSM), and chemical fertilizer application with straw biochar (NB). The chemical fertilizer comprised 150 kg ha^-1^ of nitrogen, 75 kg ha^-1^ of P_2_O_5_, and 60 kg ha^-1^ of K_2_O in the form of urea, calcium magnesium phosphate, and potassium chloride, respectively. To produce biochar, the maize straw was subjected to pyrolysis at 450°C for 48 h. The three straw treatments were annually applied at a carbon input of 1,000 kg ha^-1^ under three straw amendments. The carbon ratio of straw to pig manure applications was calculated at a 9:1 ratio based on dry matter. Monoculture maize (*Zea mays* L.) was annually sowed in April and harvested in July. The maize straw, pig manure, and straw biochar used contained 391.16 g kg^−1^, 334.05 g kg^−1^, and 530.99 g kg^−1^ of carbon, and 1.26 g kg^−1^, 16.02 g kg^−1^, and 3.26 g kg^−1^ of phosphorus, respectively. All fertilizers and organic materials were applicated to each corresponding plot before sowing.

**Soil sampling and physicochemical properties**

Soil samples gathered from each plot in 2010 before the experiments as the 0-year samples. After that samples were collected from each plot annually in late July from 2011 to 2017, after maize harvest. Ten soil cores were collected in an ‘S’-type pattern from each plot, and then mixed thoroughly to make one sample. Hence, 120 soil samples were gathered through the experimental years totally. Twenty soil cores (0−20 cm) were gathered as a composite sample per plot. The composite sample was then sealed in a sterilized polyethylene wrapper, and transported to laboratory by placed on ice within 24 hours. Soil samples were sieved (2 mm) to fully blending and remove visible stones and plant debris. Each sample was separated into two subsamples, a subsample stored at 4°C for physicochemical analysis; the other one at −80°C for extracting DNA.

Soil pH was measured by a glass electrode in a water-to-soil ratio = 2.5:1. Soil organic carbon (SOC) content was examined by wet digestion employing the potassium dichromate volumetric method [1]. Soil available nitrogen (AN) and total nitrogen (TN) were respectively determined by the alkaline hydrolysis diffusion and the micro-Kjeldahl methods [1,2]. The available phosphorus (AP) was extracted with NaHCO_3_ solution and total phosphorus (TP) was digested using HF-HClO_4_ solution, and then examined by the molybdenum-blue method [3,4]. The ratio of AP to TP represents the phosphorous activation coefficient (PAC). The available potassium (AK) extracted by CH_3_CO_2_Na solution and total potassium (TK) was digested by HF-HClO_4_ solution, then were assayed by an atomic absorption spectrophotometer [5]. Soil acid phosphomonoesterase (ACP) and ALP activities were separately measured by spectrophotometer at 405 nm absorption. The *p*-nitrophenyl phosphate (*p*-NP) solution employing as the substance with using buffer respectively adjusted to pH 6.5 and 11.0, ACP and ALP activities were expressed as mg *p*-NP g^−1^ soil h^−1^[6].

**Soil DNA extraction and *phoD* gene sequencing**

Total soil DNA was extracted using the PowerSoil DNA Isolation kit (Qiagen, Hilden, Germany) from 0.5 g soil in accordance with manufacturer’s protocol. DNA quality and quantity were examined by a NanoDrop ND-2000 spectrophotometer (NanoDrop, ND-2000, Wilmington, DE, USA). To target the ALP-producing bacterial community, the *phoD* gene was amplified using the primer set ALPS-F730 and ALPS-R1101 [7]. Amplicons obtained were pulled in equimolar ratios and sequenced based on Illumina MiSeq PE300 platform (Illumina Inc., San Diego, CA, USA). Subsequently, the raw sequences were subjected to quality trimming and demultiplexing using the UPARSE pipeline, following the Quantitative Insights into Microbial Ecology 2 pipeline [8]. The high-quality sequences with ≥ 75% of nucleotide identity were clustered into a same operational taxonomic unit (OTUs). One representative sequence per OTU was used to assign taxonomic classification through the online BLAST against in the *nt* database. To minimize the effect of sampling effort on the OTU-based analysis, the original OTU table was rarified to an equal depth of 15,230 sequences per sample. A total of 6,647 ALP-producing bacterial OTUs were identified from 1,827,600 high-quality sequences after rarefaction to an equal sequencing depth across samples.

**DNA stable isotope probing microcosm experiments**

To determine whether there was variation in the network structure of ALP-producing bacteria in response to straw amendments, we conducted a ^18^O-H_2_O DNA stable-isotopic probing (DNA-SIP) microcosm experiment. This technique utilizes ^18^O-labeled water to identify the ALP-producing bacterial interactions based on the assimilation of oxygen derived from water. Briefly, 5 g of soil samples were collected from field plots in each treatment at four time points (0, 1, 6, and 7 years). The selected samples were pre-incubated for 5 days at the room temperature. Then, 120 soil samples (5 treatments × 4 time points × 2 types of H_2_O addition × 3 replicates) were collected after incubation for 3 days at 28°C in sterilized plastic aerobic culture tubes containing 600 μl of H_2_^18^O (98% ^18^O atom) or natural abundance water (H_2_^16^O). After the incubation all samples were stored in −80°C refrigerator until DNA extraction.

The fractions of DNA in soil microcosm samples were isolated using the previously described density-gradient method [9]. To generate gradients, 3 μg DNA was added into 1.85 g ml^−1^ CsCl gradient buffer (0.1 M Tris-HCl, 0.1 M KCl, 1 mM EDTA, pH = 8.0) with a final buoyant density is 1.735 g ml^−1^. The resulting mixture then transferred into a 5 ml (13 mm × 51 mm) Quick-Seal ultracentrifuge tube, and heating sealed. The tubes were calibrated for balance and continuingly centrifuged for 44 h at 45,000 rpm using a Beckman Ultracentrifuge (Optima TLX, Beckman Coulter, Inc., Palo Alto, CA, USA). After then, the gradient solution was timely divided into 15 fractions by a calibrated infusion pump. The buoyant density of a separate fraction was detected by a digital handheld refractometer (Reichert Inc., Buffalo, NY, USA). DNA contained in the fractions were separately purified using a chromatographic column (MicroCon YM-30, Millipore) to eliminate CsCl, followed by dissolution in nuclease-free H_2_O. Copy number of the *phoD* gene in each fraction was quantified by Real-Time PCR System (CFX96 Optical, Bio-Rad Laboratories, Hercules, CA, USA). Each reaction mixture contained 10 μl TaKaRa 2× SYBR Premix Ex Taq, 0.5 μM of each the two primers, and 1 μl DNA template (1−10 ng), finally diluted into 20 μl. Three no-template reactions were constructed as blank controls in each *q*PCR run. The plasmid harboring the *phoD* gene were generated as the standard curve by 10-fold dilution. The *q*PCR runs in the program comprised 30 s at 95°C, then comprised by 40 cycles of 10 s at 95°C and 30 s at 55°C, the melt curve finally step from 72°C to 95°C. The *q*PCR for each fraction from the DNA-SIP experiments was performed in triplicate, and amplification efficiencies of > 95% were obtained with *r*^2^ values of 0.99. Standard curves for the ALP-producing bacterial community were obtained using a dilution series (10^2^ to 10^8^ copies) of plasmid DNA containing bacterial *phoD* gene fragment. The natural control of water abundance was proved to be valuable method for discerning the ‘heavy’ DNA. The abundance of ‘heavy’ fractions was significantly higher in H_2_^18^O-labled fractions than in natural control.

**Network analyses**

Networks were constructed by dividing each treatment into subsets according to four temporal stages: 0−1 year, 2−3 year, 4−5 year, and 6−7 year. Each network consisted of 6 samples (2 time points × 3 replicates). All OTUs present in more than two-thirds of the samples in each subset were retained for subsequent network analysis. Network construction was based on co-occurrence patterns of OTUs with parameters of *r* > 0.8 or −0.8 and *P* < 0.01. Various topological properties were extracted to portray the topological structure of the networks, including numbers of nodes, positive edges, negative edges, average degree, average cluster coefficient, average path length, modularity, and density. The networks were computed based on a sparCC correlation matrix using the ‘ggClusterNet’ package in R, and were visualized using Gephi software (version 0.9.1) [10,11]. Networks with higher connectivity and higher connectance usually have higher complexity, which can enhance their resistance and resilience. The overall stability of a network depends on the balance of various counteracting forces owing to variations in network topology. The values of cohesions (positive and negative) for sample *j* were calculated using the sum of significant correlations between taxa, weighted by taxa abundance as follows [12,13]: $C_{j}^{pos}=\sum_{i=1}^{n} a_{i}\cdot\bar{r}_{l, r>0}$ (positive cohesion), $C_{j}^{neg}=\sum_{i=1}^{n} a_{i}\cdot\bar{r}_{l, r<0}$(negative cohesion), where *a_i_* is the abundance of OTU *i* in sample *j*, and $\bar{r_{i}}{}_{,r>0}$ and $\bar{r_{i}}{}_{,r<0}$ are positive and negative connectedness, respectively. In the given network, the positive $\bar{r_{i}}$ (*r* > 0) or negative connectedness $\bar{r_{i}}$ (*r* < 0) for a given OTU *i* was determined based on its significant positive or negative correlations with all other nodes within a network. The range of negative and positive cohesions is −1 to 0 and 0 to 1, respectively, with the higher absolute values representing stronger or more significant correlations.

Network robustness is defined as the proportion of nodes (OTUs) persistence in a network when nodes were randomly deleted by certain proportion [14,15]. To detect the effect of randomly removed nodes on the remains, the abundance-weighted mean interaction strength (wMIS) of node *i* were calculated as follows: $\mathrm{wMIS}_{i}=\frac{\sum_{i\neq j} b_{j}s_{ij}}{\sum_{i\neq j} b_{j}}$ , where *b_j_* is the relative abundance of node *j*, *s_ij_* is the strength of association between node *i* and *j*. The robustness of a network was determined by the proportion of remaining nodes after node removal. We then calculated the average network robustness when more than 50% of nodes were randomly removed.

The node vulnerability is determined by its relative contribution to the overall network efficiency. The vulnerability of networks is calculated by the maximum vulnerability of all nodes in a given network as$max\left( \frac{E-E_{i}}{E} \right)$ , here *E* is the global network efficiency and *E_i_* is the global network efficiency if deleting node *i* and its edges. The global network efficiency was examined as the average of the efficiencies over all node pairs as $E=\frac{1}{n(n-1)}\sum_{i\neq j} \frac{1}{d(i,j)}$, where *d* (*i,j*) is the number of the shortest path among node *i* and *j*. In ecological networks, efficiency is described as the speed at which the effects of biological/ecological events are transmitted through the network.

**Statistical analyses**

One-way analysis of variance used to examine the differences in soil properties, ALP-producing bacterial diversity, enzymatic activity, and maize yield among years under different treatments finished by SPSS 21.0 (SPSS, Chicago, IL, USA). The ALP-producing bacterial diversity was represented by its Shannon index and Chao1 richness using the ‘vegan’ package in R. The multiple linear regression (finished by “lm” function of the ‘stats’ package in R) and the variance decomposition analysis (finished by “relimp” function of ‘relaimpo’ package in R) were applied to assess the importance of soil properties on the ALP-producing bacteria (total and dominant) diversity [16]. A generalized linear model was used to examine the relationships of ALP-producing bacterial diversity (Shannon index and Chao 1 richness) and network stability (network robustness, vulnerability, and NPC ratio) with time. The temporal turnover model was fit as the equation in Liang *et al.* [17], and use *b* quantifies the turnover rate. A pairwise t-test was performed after bootstrapping (999 times) to obtain the significance of the b values [17].

Levins’ niche breadth (*B_n_*) and Pianka’s niche overlap (*O_n_*) were examined using the ‘MicroNiche’ and ‘spaa’ packages [18,19]. Principal coordinate analysis and permutational multivariate analysis of variance (PERMANOVA) based on Bray-Curties dissimilarity were employed to examine differences in community structure using the ‘vegan’ package [20]. The expression patterns of the ALP-producing bacteria were grouped into various clusters using the ‘Mfuzz’ package based on fuzzy c-means clustering [21]. Considered a synergy between an a priori assumption of a certain amount of fuzziness in the dataset and the advantage of high-quality clustering. The parameter for fuzzification was set to *m* = 3 (m: the clustering corresponds to minimizing the objective function), and the number of clusters to *c* = 4-7 (c: the number of clusters) to maintain the soft clustering of ALP-producing bacterial OTUs according to the optimal clustering of different treatments.

Random forest modeling was performed to quantitatively estimate the significant predictors of PAC and maize yield, including soil properties and ALP-producing bacterial community structure. The soil properties contain pH, SOC, AN, AK, TN, and TK, while the ALP-producing bacterial community metrics included diversity (Shannon index and Chao1 richness) and network stability (robustness, vulnerability, and NPC ratio). Structural equation modeling (SEM) was established to explore the potential relationships among soil properties, ALP-producing bacterial diversity, network stability, ALP activity, PAC, and maize yield using AMOS 23.0 (AMOS IBM, USA) [22]. Based on prior knowledge and theory, we developed a conceptual model in which straw amendments regulated ALP-producing bacterial diversity and network stability, thereby influencing ALP activity, PAC, and maize yield. Soil properties with significant impacts on the increased mean square error (MSE) of PAC and maize yield were selected to construct SEMs. Accordingly, soil properties include SOC, AN, and AK, while the ALP-producing bacterial community includes diversity (Shannon index and Chao1 richness) and network stability (robustness, vulnerability, and NPC ratio).

**REFERENCS**

1. Nelson, D.W. and L. E. Sommers. 1996. “Total Carbon, Organic Carbon, and Organic Matter”. In: D. L. Sparks, A. L. Page. editors. *Methods of soil analysis part 3–chemical methods*. ASA, Inc. 1996. p. 961-1010. https://doi.org/10.2136/sssabookser5.3.

2. Bremner, J. M., C. S. Mulvaney. 1996. “Nitrogen-total.” In: D. L. Sparks, A. L. Page. editors. *Methods of soil analysis part 3–chemical methods*. ASA, Inc. p. 1085–1121. https://doi.org/10.2136/sssabookser5.3.

3. Olsen, S. R., C. V. Cole, F. S. Watanabe, L. Dean. “Estimation of available phosphorus in soils by extraction with sodium bicarbonate.” *Circular* (No. 939). Washington DC: USDA Press 1954. p. 1–19. https://api.semanticscholar.org/CorpusID:3684522.

4. O'Halloran, Ivan P., Barbara Cade-Menun. “Total and organic phosphorus. In: Carter, M. R., E. G. Gregorich. editors.” *editors Soil sampling and methods of analysis (2nd ed) part III–soil chemical analyses.* Boca Raton: CRC Press 2007. p. 267–271. https://doi.org/10.1201/9781420005271.ch24.

5. Kanehiro, Y., G. D. Sherman. “Fusion with sodium carbonate for total elemental analysis.” In: C. A. Black. editor. *Methods of soil analysis, part 2–agronomy 9*. ASA, Inc 1965. p. 952–958. https://doi.org/10.2134/agronmonogr9.2.c12.

6. Jiang, Yuji, Manqiang Liu, Jiabao Zhang, Yan Chen, Xiaoyun Chen, Lijun Chen, Huixin Li, et al. 2017. “Nematode grazing promotes bacterial community dynamics in soil at the aggregate level.” *The ISME Journal* 11: 2705–2717. https://doi.org/10.1038/ismej.2017.120.

7. Sakurai, Michihiko, Jun Wasaki, Yuiko Tomizawa, Takuro Shinano, Mitsuru Osaki. 2008. “Analysis of bacterial communities on alkaline phosphatase genes in soil supplied with organic matter.” *Soil Science and Plant Nutrition* 54: 62–71. https://doi.org/ 10.1111/j.1747-0765.2007.00210.x.

8. Bolyen, Evan, Jai Ram Rideout, Matthew R. Dillon, Nicholas A. Bokulich, Christian C Abnet, Gabriel A Al-Ghalith, Harriet Alexander, et al. 2019. “Reproducible, interactive, scalable and extensible microbiome data science using QIIME 2.” *Nature Biotechnology* 7: 335–336. https://doi.org/10.1038/s41587-019-0209-9.

9. Blazewicz, Steven J., Bruce A. Hungate, Benjamin J. Koch, Erin E. Nuccio, Ember Morrissey, Eoin L. Brodie, Egbert Schwartz, et al. 2020. “Taxon-specific microbial growth and mortality patterns reveal distinct temporal population responses to rewetting in a California grassland soil.” *The ISME Journal* 14: 1520–1532. https://doi.org/10.1038/s41396-020-0617-3.

10. Crow, Susan E., Kate Lajtha, Timothy R. Filley, Christopher W. Swanston, Richard D. Bowden, Bruce A. Caldwell. 2009. “Sources of plant-derived carbon and stability of organic matter in soil: implications for global change.” *Global Change Biology* 15: 2003–2019. https://doi.org/ 10.1111/j.1365-2486.2009.01850.x.

11. Tisdall, J. M., J. M. Oades. 1982. “Organic matter and water-stable aggregates in soils.” *European Journal of Soil Science* 33: 141–163. https://doi.org/10.1111/j.1365-2389.1982.tb01755.x.

12.  Hernandez, Damian J., Aaron S. David, Eric S. Menges, Christopher A. Searcy, and Michelle E. Afkhami. 2021. “Environmental stress destabilizes microbial networks.” *The ISME Journal* 15: 1722–1734. https://doi.org/10.1038/s41396-020-00882-x.

13. Herren Cristina M., Katherine D. McMahon. 2017. “Cohesion: A method for quantifying the connectivity of microbial communities.” *The ISME Journal* 11: 2426–2438. https://doi.org/ 10.1038/ismej.2017.91.

14. Yuan, Mengting M. Xue Guo, Linwei Wu, Ya Zhang, Naijia Xiao, Daliang Ning, Zhou Shi, et al. 2021. “Climate warming enhances microbial network complexity and stability.” *Nature Climate Change* 11: 343–348. https://doi.org/ 10.1038/s41558-021-00989-9.

15. Montesinos-Navarro, Alicia, Fernando Hiraldo, José L. Tella, and Guillermo Blanco. 2017. “Network structure embracing mutualism–antagonism continuums increases community robustness.” *Nature Ecology and Evolution* 1: 1661–1669. https://doi.org/ 10.1038/s41559-017-0320-6.

16. Groemping Ulrike. 2006. “Relative importance for linear regression in R: The package relaimpo.” *Journal of Statistical Software* 17: 1–27. https://doi.org/ 10.18637/jss.v017.i01.

17. Liang, Yuting, Yuji Jiang, Feng Wang, Congqing Wen, Ye Deng, Kai Xue, Yujia Qin, et al. 2015. “Long-term soil transplant simulating climate change with latitude significantly alters microbial temporal turnover.” *The ISME Journal* 9: 2561–2572. https://doi.org/ 10.1038/ismej.2015.78.

18. Finn, D. R., J. Yu, Z. E. Ilhan, V. M. C. Fernandes, C. R. Penton, R. Krajmalnik-Brown, F. Garcia-Pichel, et al. 2020. “MicroNiche: an R package for assessing microbial niche breadth and overlap from amplicon sequencing data.”  *FEMS Microbiology Ecology*  96: fiaa131. https://doi.org/10.1093/femsec/fiaa131.

19. Zhang, Jinlong, Keping Ma. “spaa: An R package for Computing Species Association and Niche Overlap.” 2013. *Research Progress of Biodiversity Conservation in China* (Volume X), pp 165–174.

20. Dixon, Phillip. 2003. “VEGAN, a package of R functions for community ecology.” *Journal of Vegetation Science* 14:927–930. https://doi.org/10.1111/j.1654-1103.2003.tb02228.x.

21. Kumar, Lokesh, Matthias E. Futschik. 2007. “Mfuzz: A software package for soft clustering of microarray data.” *Bioinformation* 2: 5–7. https://doi.org/10.6026/97320630002005.

22.  Chen, Lijun, Yuji Jiang, Chao Liang, Yu Luo, Qingsong Xu, Cheng Han, Qiguo Zhao, Bo Sun. 2019. “Competitive interaction with keystone taxa induced negative priming under biochar amendments.” *Microbiome*, 7(1), 77. https://doi.org/10.1186/s40168-019-0693-7.


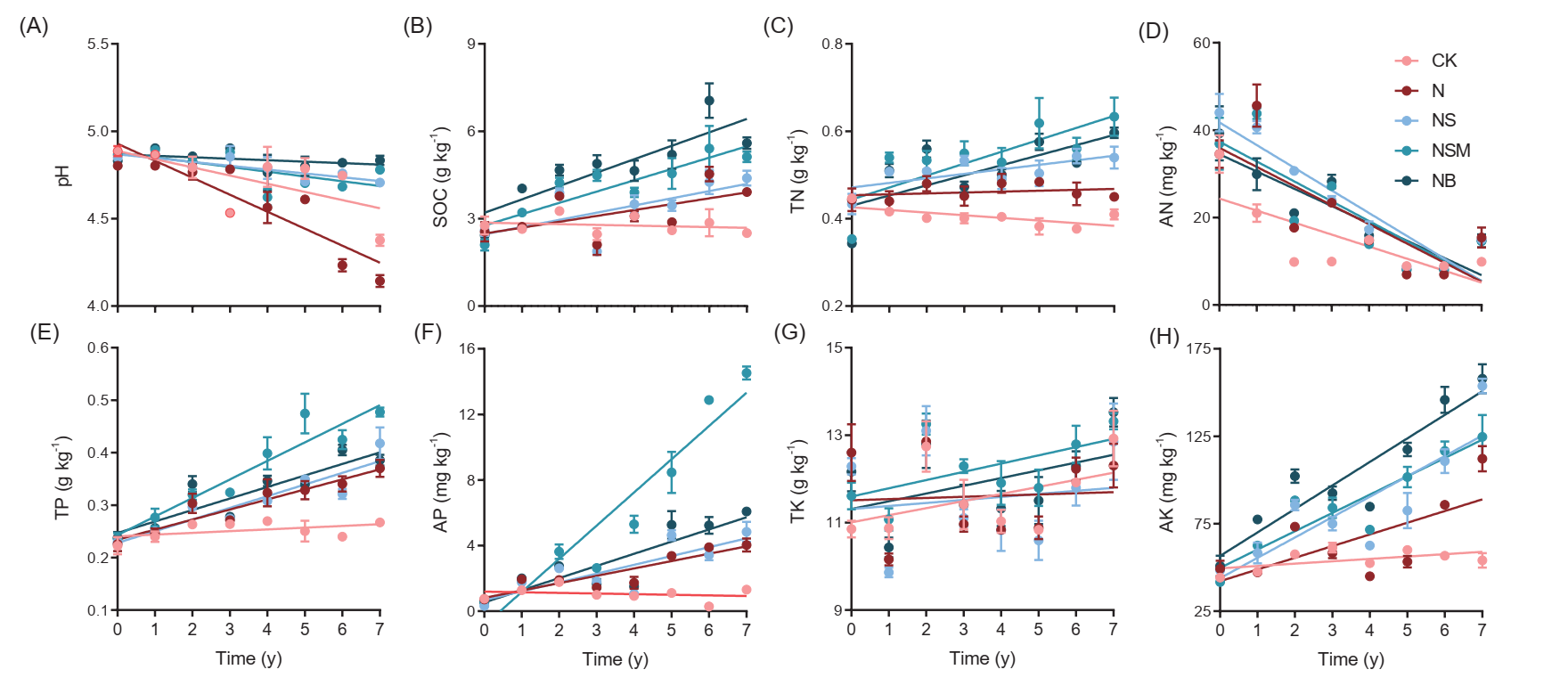


**Figure S1 Temporal dynamics of soil chemical properties over time under different** **fertilization treatments.** (A-H) Temporal dynamic of soil pH (A), organic carbon (B, SOC), total nitrogen (C, TN), total phosphorus (D, TP), total potassium (E, TK), available phosphorus (F, AP), available nitrogen (G, AN), and available potassium (H, AK). CK, no fertilizer; N, NPK fertilizer; NS, NPK fertilizer application with straw; NSM, NPK fertilizer application with straw and pig manure; NB, NPK fertilizer application with straw biochar.


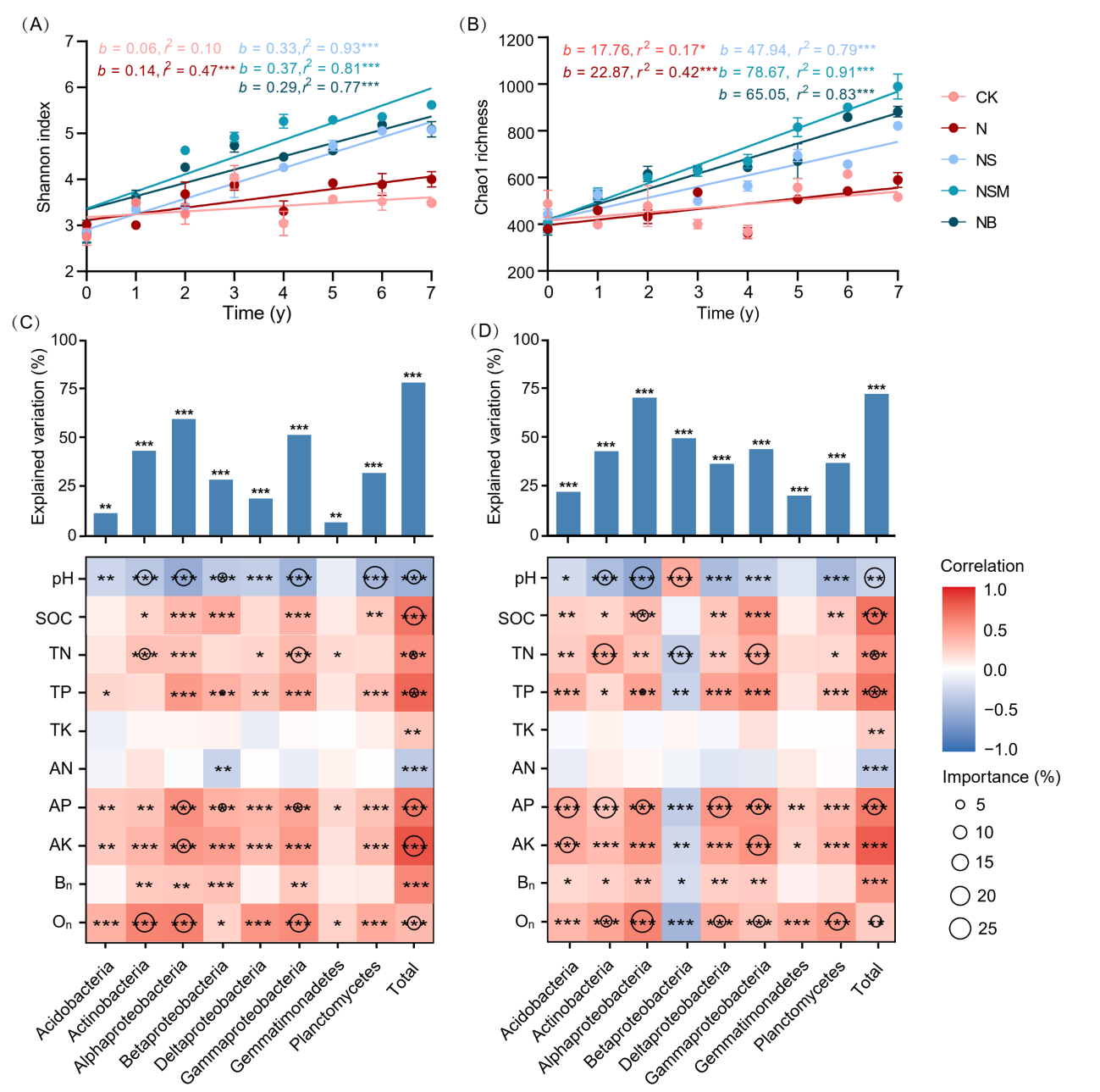


**Figure S2 Temporal dynamics of ALP-producing bacterial diversity over time under different treatments.** (A, B) The line regression between the ALP-producing bacterial Shannon index (A) and Chao1 richness (B) over time. (C, D) The importance of soil properties, niche breadth (*B_n_*), and niche overlap (*O_n_*) on the Shannon index (C) and Chao1 richness (D) of the whole and dominant phyla/classes of ALP-producing bacterial community across different treatments. *b*, the slope of line regression. SOC, soil organic carbon; TN, total nitrogen; TP, total phosphorus; TK, total potassium; AN, available nitrogen; AP, available phosphorus; AK, available potassium. CK, no fertilizer; N, NPK fertilizer; NS, NPK fertilizer application with straw; NSM, NPK fertilizer application with straw and pig manure; NB, NPK fertilizer application with straw biochar. * *p* < 0.05, ** *p* < 0.01, *** *p* < 0.001.


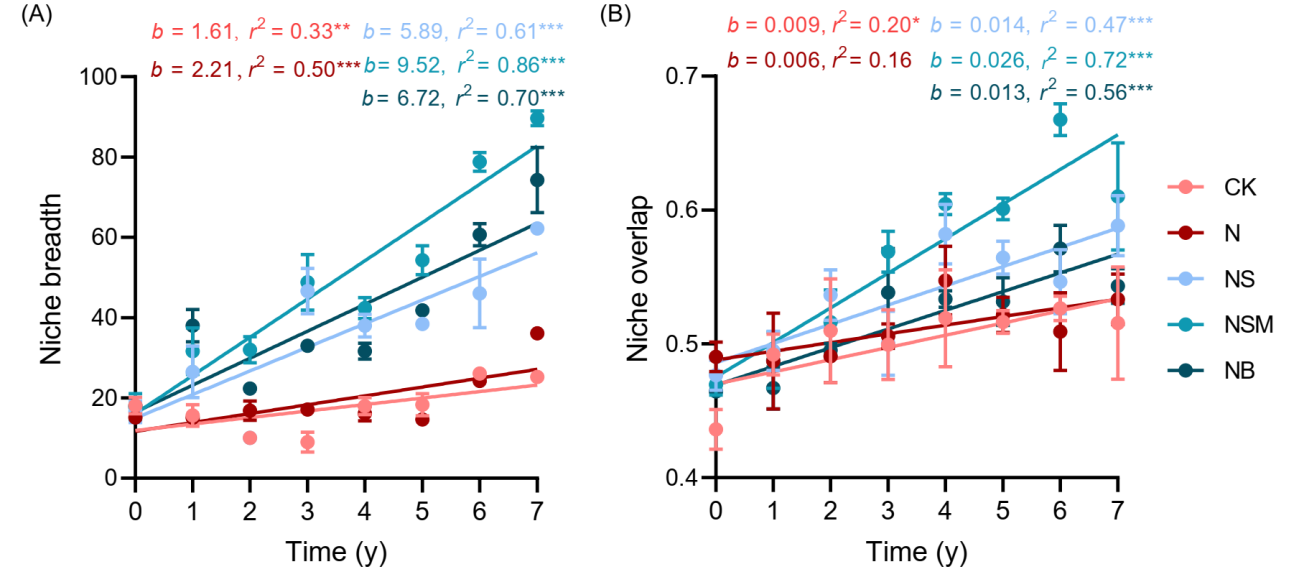


**Figure S3** **Temporal dynamics of niche breadth index and niche overlap index over time under fertilization different treatments.** (A) Temporal dynamic of ALP-producing bacterial niche breadth; (B) Temporal dynamic of ALP-producing bacterial nicheoverlaps. *b*, the slope of line regression. CK, no fertilizer; N, NPK fertilizer; NS, NPK fertilizer application with straw; NSM, NPK fertilizer application with straw and pig manure; NB, NPK fertilizer application with straw biochar. * *p* < 0.05, ** *p* < 0.01, *** *p* < 0.001.


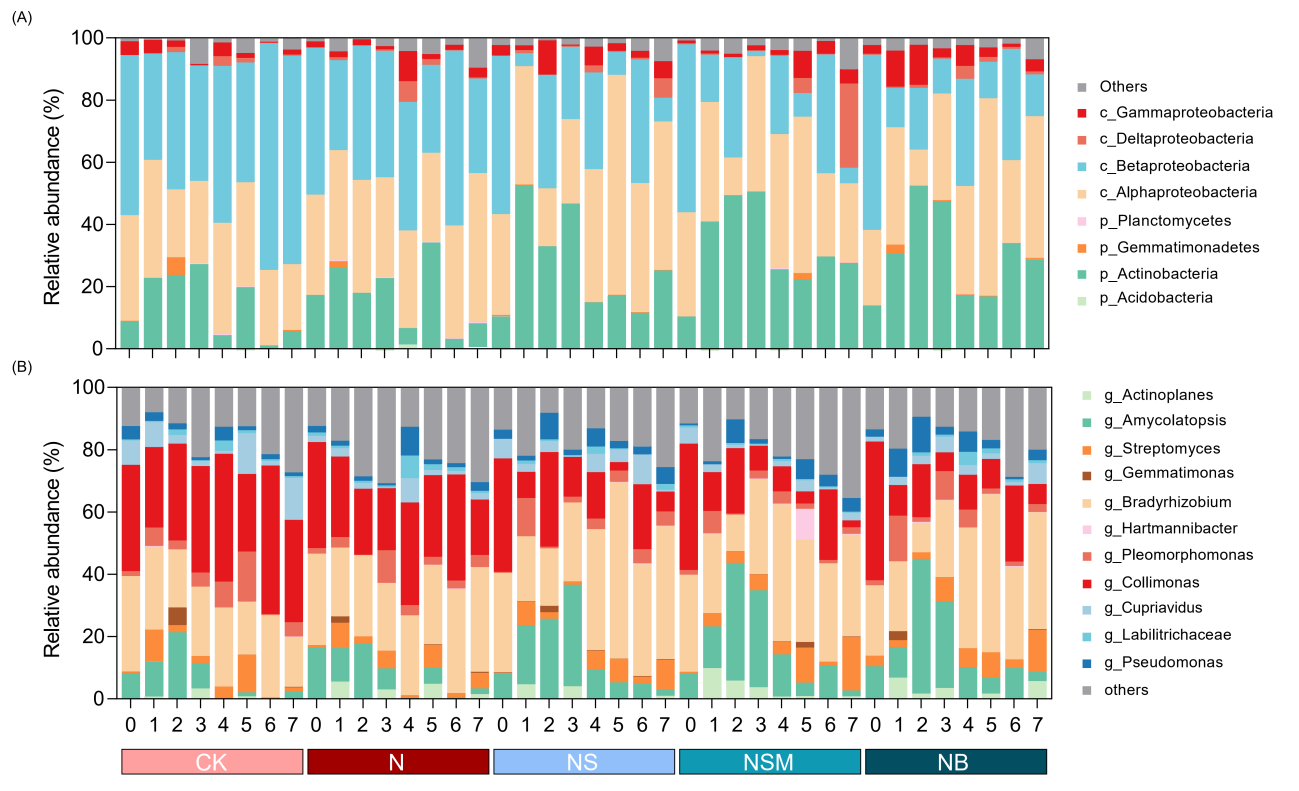


**Figure S4** **Relative abundance of alkaline phosphomonoesterase (ALP) producing bacterial community over time under different treatments.** (A, B) Taxonomic compositions of ALP-producing bacteria communities at phylum (A) and genus (B) level. CK, no fertilizer; N, NPK fertilizer; NS, NPK fertilizer application with straw; NSM, NPK fertilizer application with straw and pig manure; NB, NPK fertilizer application with straw biochar.


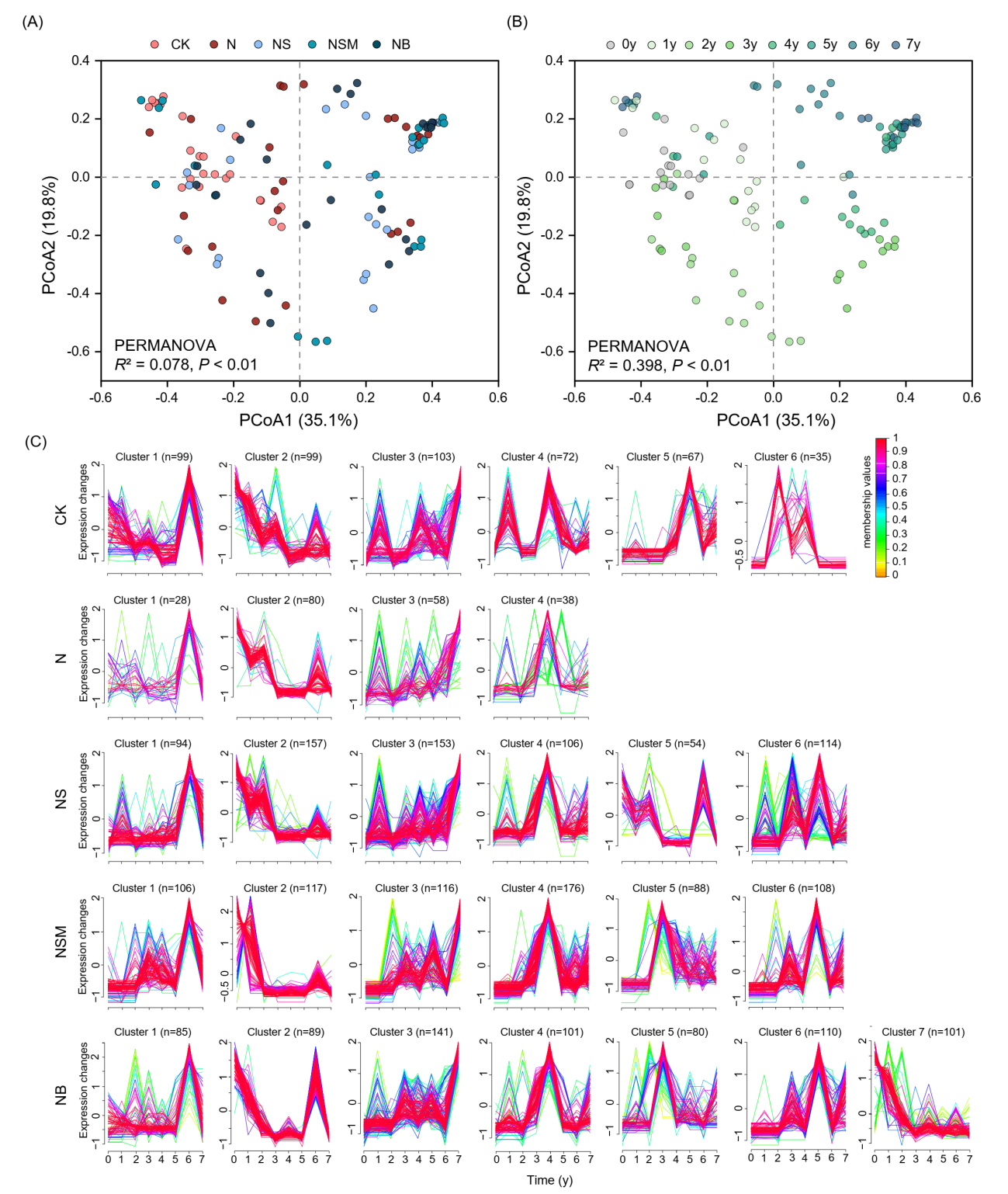


**Figure S5** **The structure of alkaline phosphomonoesterase (ALP) producing bacterial community.** (A, B) Principal coordinate analysis (PCoA) of ALP-producing bacterial community under different fertilization treatments (A) and at different time points (B) based on the sequencing profiles of *phoD* gene. (C) All the OTUs present in more than 50% of the samples were used for Mfuzz analysis. The x-axis represents different time points, and the y-axis represents the standardized expression change. OTUs within the same cluster indicate that their pattern of change are similar on an annual time scale. CK, no fertilizer; N, NPK fertilizer; NS, NPK fertilizer application with straw; NSM, NPK fertilizer application with straw and pig manure; NB, NPK fertilizer application with straw biochar.


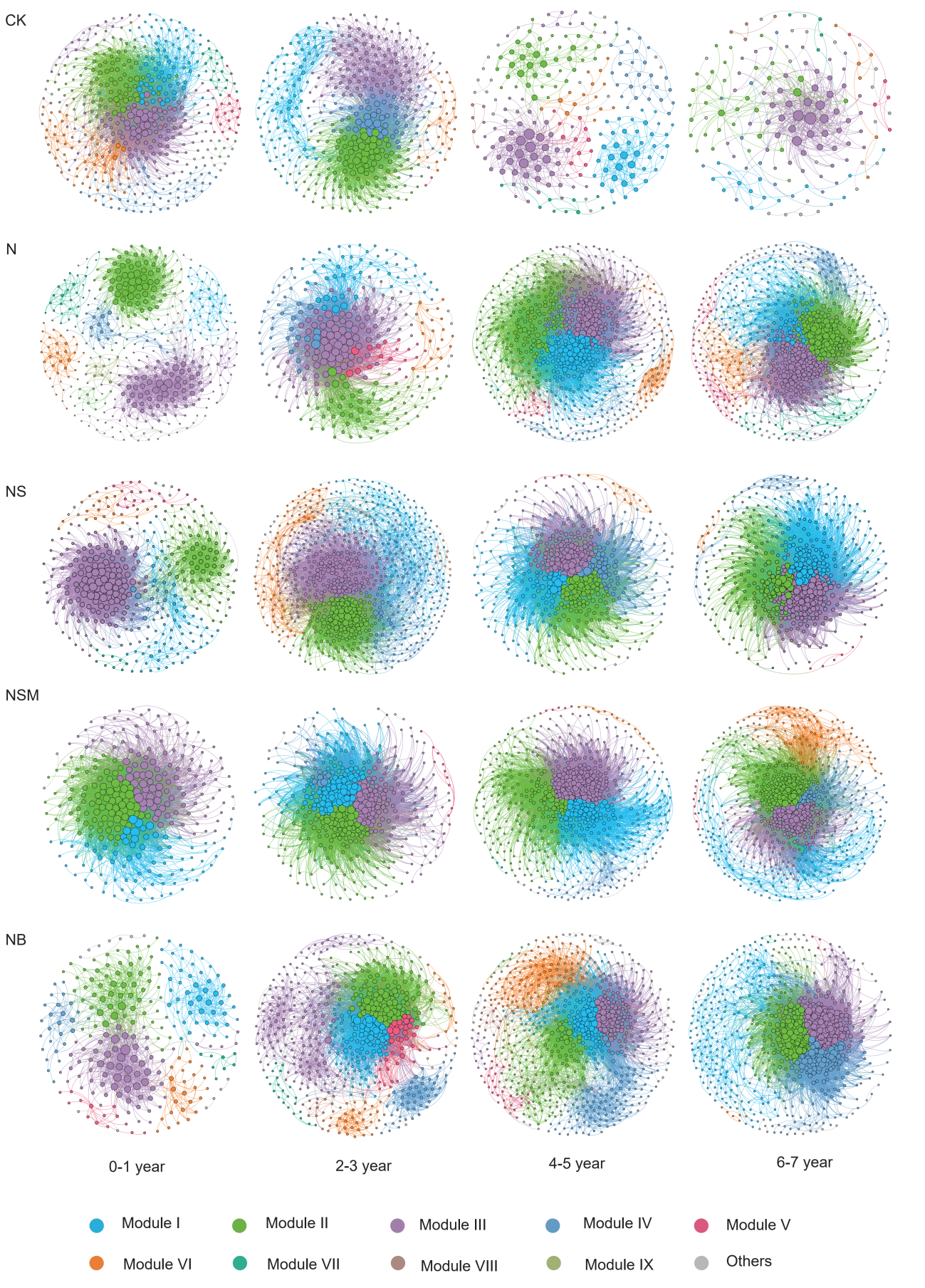


**Figure S6** **Temporal dynamics of alkaline phosphomonoesterase (ALP) producing bacterial networks.** Dynamic of soil ALP-producing bacterial networks. The networks constructed based at four time points (0−1 year, 2−3 year, 4−5 year, and 6−7 year) under each treatment. A connection stands for a strong (Spearman’s *r* > 0.8 or < −0.8) and significant (*P* value < 0.01) correlation for the non-amended and straw-amended treatments. The size of each node is proportional to the number of connections. The nodes and edges were colored by module clusters. CK, no fertilizer; N, NPK fertilizer; NS, NPK fertilizer application with straw; NSM, NPK fertilizer application with straw and pig manure; NB, NPK fertilizer application with straw biochar.


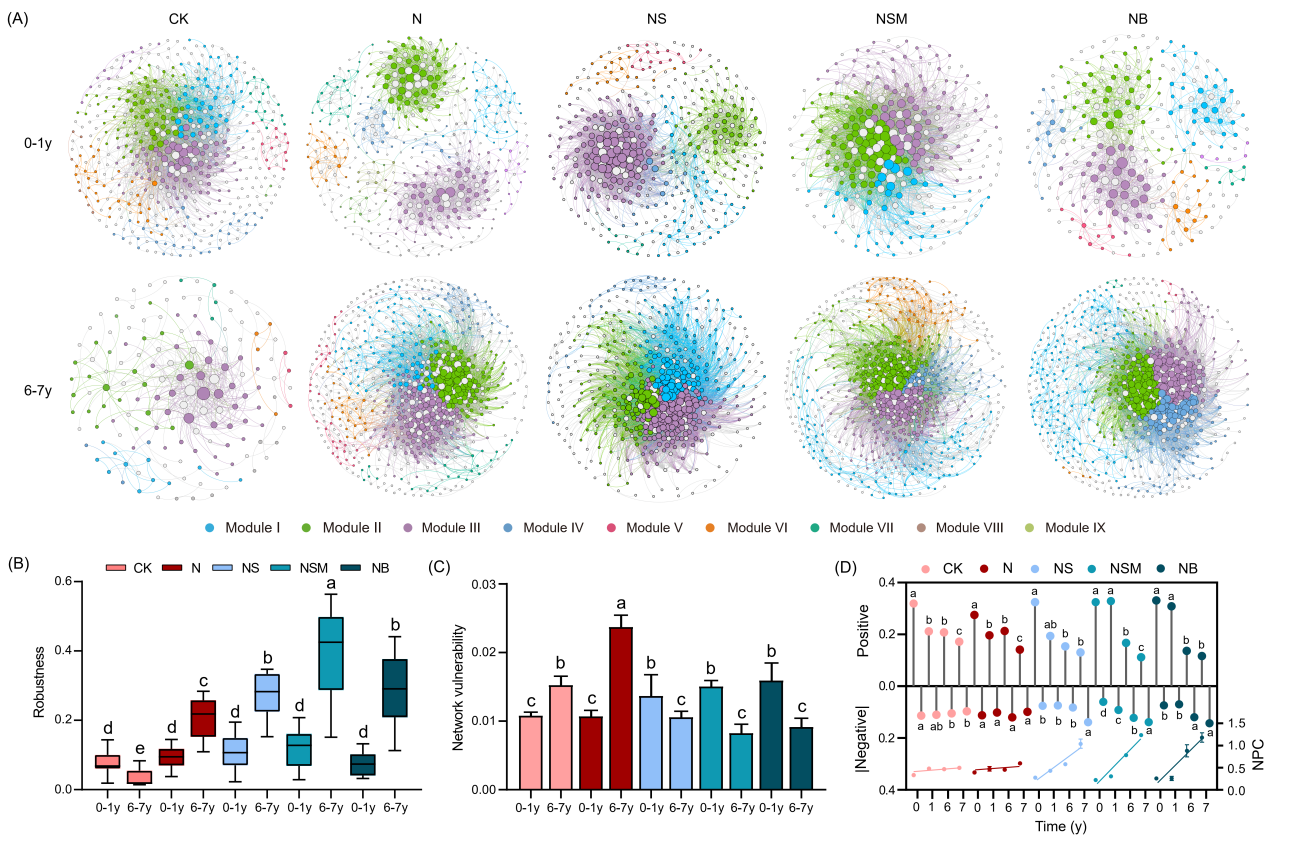


**Figure S****7 The co-occurrence patterns and network stability of soil alkaline phosphomonoesterase (ALP) producing bacteria under fertilization treatments at 0-1 year and 6-7 year in DNA-SIP microcosm experiments.** (A) ALP-producing bacterial networks. (B) Robustness. (C) Vulnerability. (D) Positive cohesions, negative cohesions and the ratios of negative cohesion to positive cohesion. Bars with different lowercase letters are significantly (*p* < 0.05) different under fertilization treatments at each time point by Tukey’s post hoc tests. CK, no fertilizer; N, NPK fertilizer; NS, NPK fertilizer application with straw; NSM, NPK fertilizer application with straw and pig manure; NB, NPK fertilizer application with straw biochar. * *p* < 0.05, ** *p* < 0.01, *** *p* < 0.001.

**
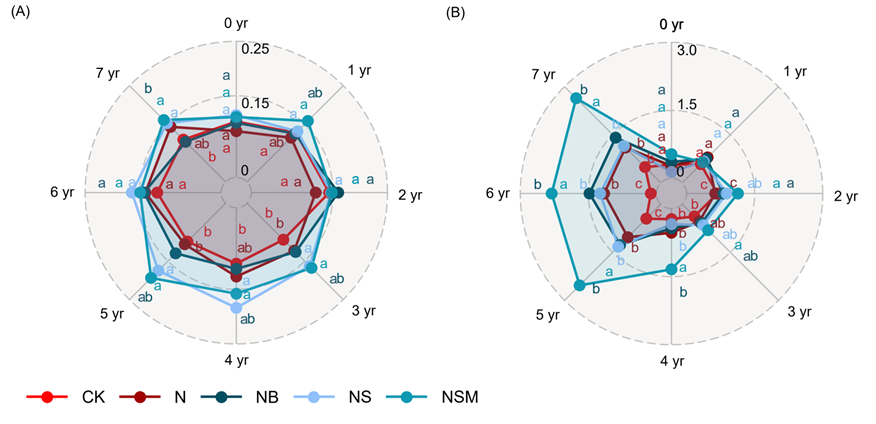
Figure S8 Temporal dynamics of soil acid phosphomonoesterase (ACP) and phosphorus activation coefficient (PAC).** (A) Temporal dynamics of soil ACP actvity; (B) Temporal dynamics of soil PAC under different treatment. Lowercase letters are significantly (*p* < 0.05) different between treatments in the same year by Tukey’s post hoc tests. CK, no fertilizer; N, NPK fertilizer; NS, NPK fertilizer application with straw; NSM, NPK fertilizer application with straw and pig manure; NB, NPK fertilizer application with straw biochar.


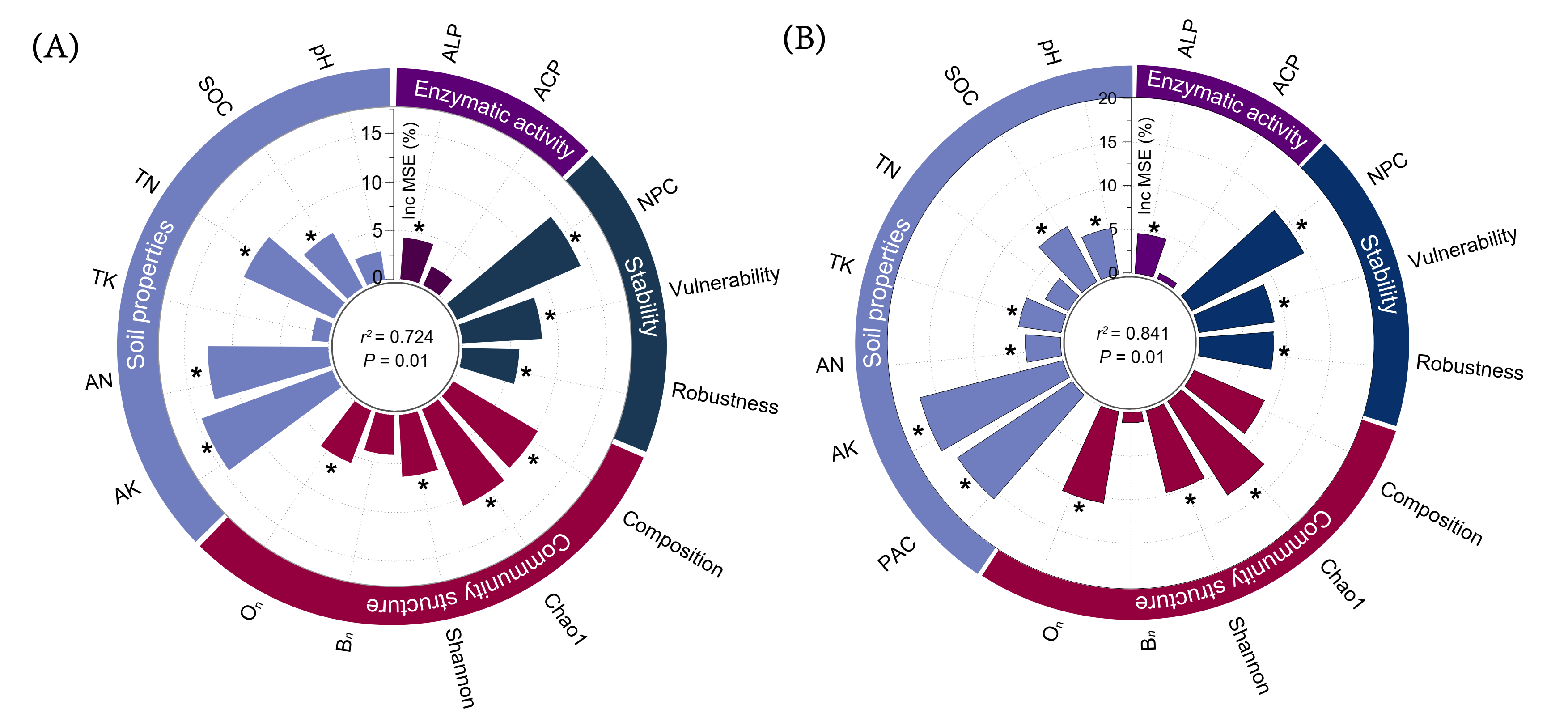


**Figure S9** **Random forest model on soil phosphorus activation coefficient and maize yields.** (A, B) Mean contribution (% of increased mean square error, MSE) of soil abiotic and biotic properties on soil phosphorus activation coefficient (A, PAC) and maize yield (B) based on random forest modeling. Soil properties include pH, soil organic carbon (SOC), total nitrogen (TN), total phosphorus (TP), total potassium (TK), available nitrogen, available phosphorus (TP), and available potassium (TK). The alkaline phosphomonoesterase (ALP) producing community includes diversity (Shannon index and Chao1 richness), and structure (first principal coordinates, PCoA1), and network stability (robustness, vulnerability, and NPC ratio), niche breadth index (*B_n_*) and niche overlap index (*O_n_*), acid and alkaline phosphomonoesterase (ACP and ALP) activities. NPC, the ratios of negative cohesion to positive cohesion. * *p* < 0.05.


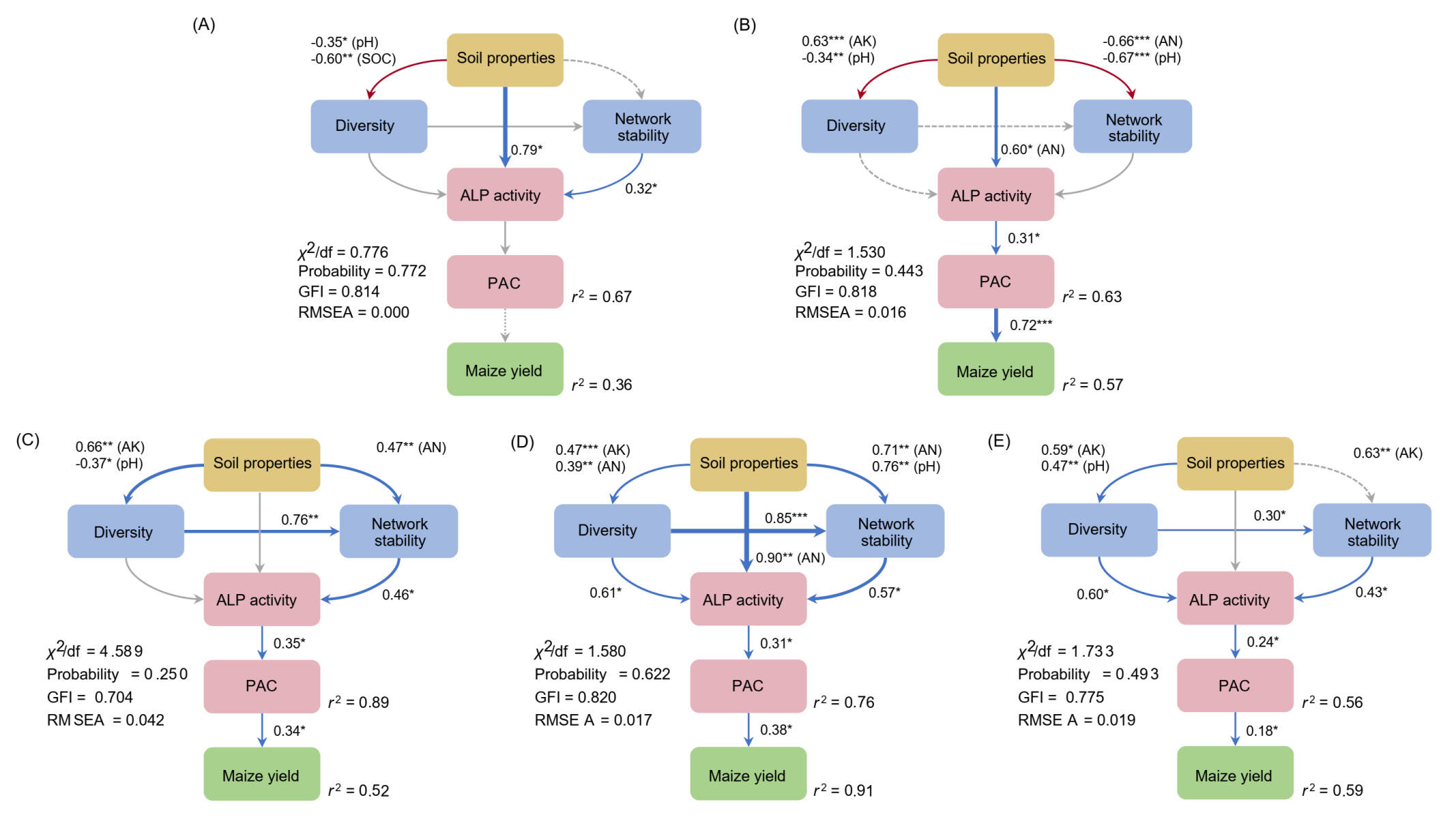


**Figure S****10 The structural equation modeling under different treatment.** (A-E) CK (A), N (B), NS (C), NSM (D), and NB (E) treatments. The proportion of explained variance (R^2^) appears alongside PAC and maize productivity in the model. Soil properties include pH, soil organic carbon (SOC), total nitrogen (TN), total phosphorus (TP), total potassium (TK), available nitrogen (AN), available phosphorus (AP), and available potassium (AK), while the ALP-producing bacterial community includes diversity (Shannon index and Chao1 richness) and network stability (robustness, vulnerability, and NPC ratio). Blue and red lines indicate positive and negative effects, respectively. The width of the arrows indicates the strength of significant standardized path coefficients. Paths with non-significant coefficients are presented as gray lines. CK, no fertilizer; N, NPK fertilizer; NS, NPK fertilizer application with straw; NSM, NPK fertilizer application with straw and pig manure; NB, NPK fertilizer application with straw biochar. * *p* < 0.05, ** *p* < 0.01, *** *p* < 0.001.


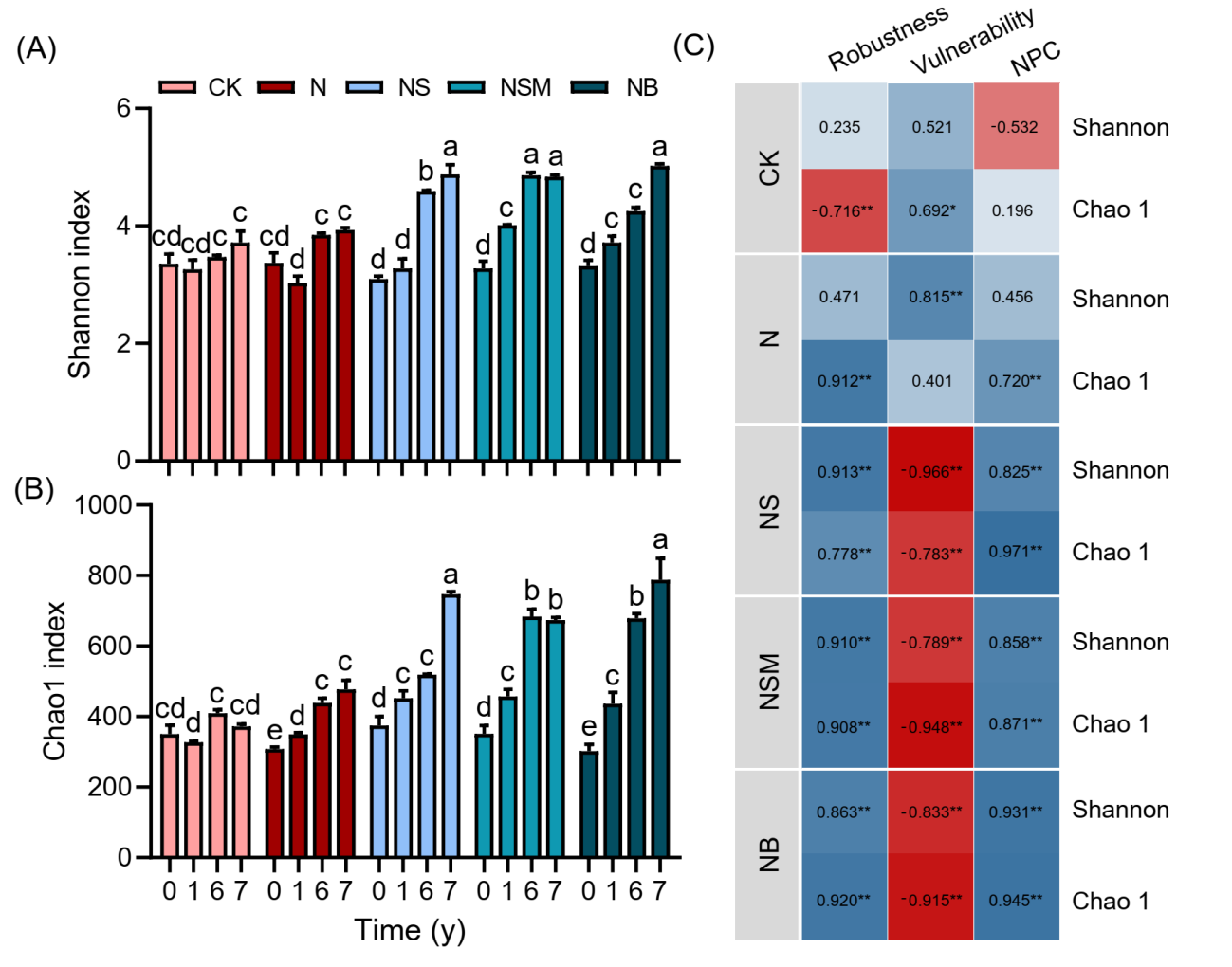


**Figure S11** **The diversity of soil alkaline phosphomonoesterase (ALP) producing bacteria and its correlations with network stability in DNA-SIP microcosm experiments.** (A) Shannon index. (B) Chao1 richness. (C) The relationships of network stability (robustness, vulnerability, and NPC) with Shannon index and Chao1 richness. Bars with different lowercase letters are significantly (*P* < 0.05) different under fertilization treatments at each time point by Tukey’s post hoc tests. NPC, the ratios of negative cohesion to positive cohesion. CK, no fertilizer; N, NPK fertilizer; NS, NPK fertilizer application with straw; NSM, NPK fertilizer application with straw and pig manure; NB, NPK fertilizer application with straw biochar. * *p* < 0.05, ** *p* < 0.01, *** *p* < 0.001.
